# Identification of New Antibodies Targeting Malignant Plasma Cells for Immunotherapy by Next-Generation Sequencing-Assisted Phage Display

**DOI:** 10.3389/fimmu.2022.908093

**Published:** 2022-06-17

**Authors:** Steffen Krohn, Ammelie Svea Boje, Carina Lynn Gehlert, Sebastian Lutz, Nikos Darzentas, Henrik Knecht, Dietrich Herrmann, Monika Brüggemann, Axel J. Scheidig, Katja Weisel, Martin Gramatzki, Matthias Peipp, Katja Klausz

**Affiliations:** ^1^Division of Antibody-Based Immunotherapy, Department of Internal Medicine II, University Hospital Schleswig-Holstein and Christian-Albrechts-University Kiel, Kiel, Germany; ^2^Unit for Hematological Diagnostics, Department of Medicine II, University Hospital Schleswig-Holstein and Christian-Albrechts-University Kiel, Kiel, Germany; ^3^Zoological Institute, Christian-Albrechts-University of Kiel, Kiel, Germany; ^4^Department of Oncology, Hematology, Bone Marrow Transplant (BMT) with Section of Pneumology, University Medical Center Hamburg-Eppendorf (UKE), Hamburg, Germany

**Keywords:** immunotherapy, multiple myeloma, monoclonal antibody, CD38, ICAM-1/CD54, phage display, cellular panning, next-generation sequencing – NGS

## Abstract

To identify new antibodies for the treatment of plasma cell disorders including multiple myeloma (MM), a single-chain Fragment variable (scFv) antibody library was generated by immunizing mice with patient-derived malignant plasma cells. To enrich antibodies binding myeloma antigens, phage display with cellular panning was performed. After depleting the immune library with leukocytes of healthy donors, selection of antibodies was done with L-363 plasma cell line in two consecutive panning rounds. Monitoring the antibodies’ enrichment throughout the panning by next-generation sequencing (NGS) identified several promising candidates. Initially, 41 unique scFv antibodies evolving from different B cell clones were selected. Nine of these antibodies strongly binding to myeloma cells and weakly binding to peripheral blood mononuclear cells (PBMC) were characterized. Using stably transfected Chinese hamster ovary cells expressing individual myeloma-associated antigens revealed that two antibodies bind CD38 and intercellular adhesion molecule-1 (ICAM-1), respectively, and 7 antibodies target yet unknown antigens. To evaluate the therapeutic potential of our new antibodies, in a first proof-of-concept study the CD38 binding scFv phage antibody was converted into a chimeric IgG1. Further analyses revealed that #5-CD38-IgG1 shared an overlapping epitope with daratumumab and isatuximab and had potent anti-myeloma activity comparable to the two clinically approved CD38 antibodies. These results indicate that by phage display and deep sequencing, new antibodies with therapeutic potential for MM immunotherapy can be identified.

## Introduction

Multiple myeloma (MM) is the second most prevalent hematological disease in industrialized countries (1). MM is characterized by malignant plasma cells located in bone marrow (BM) niches providing a microenvironment partially protecting them from therapeutic intervention. Growth and dissemination of the tumor cells to various sites in the BM and even to extramedullary sites or the blood leads to the typical symptomatic features of MM with progressive bone destruction, anemia, high blood calcium levels and/or acute renal failure (2). In the past two decades, several novel treatment options have emerged leading to a marked increase in prognosis of MM patients. One of the recent key milestones was the development of monoclonal antibodies targeting MM cells. With the approval of the first monoclonal antibodies daratumumab and elotuzumab, targeting CD38 and SLAMF7 (CD319/CS1), respectively, in 2015 and their use in various combination regimens with proteasome inhibitors, immunomodulatory drugs and chemotherapeutic agents, long-term outcome and survival of patients were substantially improved (3). The success of both monoclonal antibodies prepared the ground for immunotherapy in MM treatment and led to recent approval of the antibody-drug conjugate (ADC) belantamab mafodotin and the chimeric antigen receptor (CAR) T cell product idecabtagene vicleucel, both directed against the B cell maturation antigen (BCMA/CD269), which is exclusively expressed on healthy and malignant plasma cells (4, 5). Several other immunotherapeutic approaches including bispecific antibodies targeting BCMA are underway (6). Despite the overall improvement in patients’ outcome, myeloma remains an incurable, highly heterogeneous disease. Especially, specific subgroups of patients still have an impaired prognosis, such as frail patients, patients with high-risk cytogenetics and patients refractory to available therapeutic options (7). This highlights an unmet medical need for identifying novel targets and the development of efficient treatments for MM patients.

Phage display is a valuable technique to isolate antibodies against target structures on cell surface of tumor cells, which might have potential as new immunotherapeutic agents. In this regard, cellular panning approaches were already successfully used to identify novel antibodies targeting so far unknown tumor-associated antigens like the C-type lectin-like molecule-1 on acute myeloid leukemia (AML) cells or tumor-specific peptides of oncogenes (*RAS* and *TP53*) presented on human leukocyte antigens (HLA) (8–10). Using phage display, antibodies can be selected from synthetic, semi-synthetic, naïve or immune libraries. To enable identification of antibodies with desired binding properties, synthetic, semi-synthetic and naïve libraries have to be very large to achieve the required high initial diversity. For instance, the naïve human antibody libraries HAL9/10, the semi-synthetic n-CoDeR and the synthetic HuCAL PLATINUM libraries have theoretical diversities of 1.5 x 10^10^, 2 x 10^9^ and 4.5 x 10^10^ clones, respectively (11–13). Although specific binders can be isolated, additional *in vitro* affinity maturations can become necessary to generate high-affinity binders from these types of libraries (14). Since immune libraries are generated from immunized individuals, the embodied antibodies already passed affinity maturation, increasing the possibility to identify specific and high-affinity antibodies (15–17). Blood, BM, lymphoid organs or spleens containing plasma or B cells can be used to generate immune libraries. Of note, utilization of spleens from immunized mice for immune phage library generation is thereby as efficient as using mouse BM plasma cells for antibody discovery (18).

Today, the combination of phage display and next-generation sequencing (NGS) allows deep evaluation of newly generated antibody libraries and monitoring of panning processes. By using modern NGS platforms, several millions of variable (V) regions defining the binding specificity of an antibody can be sequenced simultaneously and potentially cover all antibodies present within an antibody library. In 2010, Ravn and colleagues were the first who defined the complementary determining region 3 of the heavy chain (CDRH3) of a synthetic antibody library by Illumina sequencing. They observed an enrichment of individual CDRH3 sequences after three rounds of panning and used the most common ones for rescue PCR and scFv antibody generation to identify potent antigen binders (19). Later, novel antibodies with therapeutic potential and high binding specificity were successfully isolated from human, mouse and rabbit synthetic, semi-synthetic, naïve and immune libraries by deep sequencing (18, 20, 21). Furthermore, combining NGS analysis of antibody repertoires and phage display helps to exploit the full potential of phage libraries and to isolate rare and diverse antibodies – with rare clones occasionally showing even higher affinity and target specificity than high-frequent ones (18, 20, 22).

In previous works, we already identified an intercellular adhesion molecule-1 (ICAM-1/CD54) antibody by cellular panning of the synthetic human Tomlinson library that had potent anti-myeloma activity *in vitro* and *in vivo* (23, 24). In this work, we immunized mice with patient-derived malignant plasma cells to generate an immune library as a source of novel anti-myeloma antibodies. Cellular panning to enrich myeloma binders in combination with NGS analyses of antigen-binding IGHV-IGHD-IGHJ rearrangements was performed. Exemplified testing of 66 randomly selected scFv phage antibodies after two rounds of panning identified 41 clonotypes derived from individual B cells including a novel CD38 and ICAM-1 antibody. In addition, NGS data revealed that further interesting antibodies might not be characterized yet. Initial functional proof-of-concept studies performed with chimeric #5-CD38-IgG1 antibody generated from a novel CD38 binding scFv phage antibody demonstrated high affinity binding and cytotoxic activity comparable to daratumumab and isatuximab against myeloma cells.

## Material and Methods

### Cell Separation

Pleural effusion and BM mononuclear cells (MNC) from plasma cell leukemia (PCL)/MM patients and PBMC from healthy donors were isolated by density gradient centrifugation and leukocytes from healthy donors were generated by two osmotic erythrocyte lysis according to standard procedures. The experiments involving human participants were reviewed and approved by the Ethics Committee of the Christian-Albrechts-University, Kiel, Germany (D408/08 and D467/15) in accordance with the Declaration of Helsinki. The patients/participants provided their written informed consent to participate in this study.

### Culture of Eukaryotic Cells

L-363, U-266, Daudi, Raji and CHO-K1 cells were obtained from the German Collection of Microorganisms and Cell Cultures (DSMZ; cat. no. ACC 49, ACC 9, ACC 78, ACC 110), whereas CEM were purchased from the American Type Culture Collection (ATCC; cat. no. CCL-119). L-363, U-266, Raji, Daudi and CEM cells were cultured in RPMI 1640 GlutaMAX medium containing 10% FCS, 1% penicillin and streptomycin (R10+; all Thermo Fisher Scientific; cat. no. 72400-021, 10270-106, 15140-122). CHO-K1 cells were cultured in DMEM medium containing 10% FCS, 1% penicillin and streptomycin (D10+; all Thermo Fisher Scientific; cat. no. 41965-039, 10270-106, 15140-122). HUVEC and CHO-S cells were ordered from Lonza (cat. no. CC-2519) and Thermo Fisher Scientific (cat. no. R800-07), respectively, and cultured according to manufacturer’s instructions. The human plasma cell line INA-6 was cultured in RPMI 1640 GlutaMAX medium containing 20% FCS, 1% penicillin and streptomycin supplemented with 10 ng/ml recombinant human IL-6 (Thermo Fisher Scientific; cat. no. PHC0061) (25). Stable transfected CHO-K1 cells expressing myeloma antigens were generated and cultured as described (23).

### Generation of Murine scFv Antibody Library

The animal experiment was reviewed and approved by the Christian-Albrechts-University Kiel along with the German Animal Protection Law. For immunization, 1 x 10^6^ PBMC from a PCL patient (95% CD38^+^/CD138^+^ plasma cells) were injected 4 times (1^st^ boost at day 14, 2^nd^ boost at day 35, 3^rd^ boost at day 56) intraperitoneally in BALB/c mice (Charles River Laboratories). 7 days after the final boost, mice were sacrificed and spleens were cryopreserved. Total RNA from spleen of the mouse with the highest antibody titer (**Supplementary Figure S1**) was prepared using TRIzol (Thermo Fisher Scientific; cat. no. 15596026) and quality controlled by agarose-formaldehyde gel electrophoresis with 2 µg total RNA (**Supplementary Figure S2**). 10 µg of total RNA were used for cDNA synthesis using oligo(dT)_15_ primer and reverse transcriptase Superscript III (Thermo Fisher Scientific; cat. no. 10368252) according to manufacturer’s instructions. Mouse variable heavy chain (VH) and variable light chain (VL) regions were amplified by PCR with degenerated primer mixes binding mouse V and J genes (**Supplementary Table 1**) and cloned into pUC19-MSC2017 plasmid (**Supplementary Figure S3**). Subsequently, VH and VL were assembled to scFv randomly prior cloning into pJB12 phagemid (kindly provided by Andreas Plückthun, University of Zurich, Switzerland) to generate the scFv antibody immune library (**Supplementary Figure S2**) (26). Details are described in the **Supplementary Information**.

### Phage Display Using Cellular Panning

500 ml medium (SB medium containing 30 g/l tryptone, 20 g/l yeast extract, 10 g/l MOPS (pH 7.0) supplemented with 1% glucose, 30 μg/ml chloramphenicol and 10 μg/ml tetracycline; all Carl Roth; cat. no. 8952.2, 2363.2, 6979.2, X997.2, 3886.3, 0237.3) were inoculated with *E. coli* containing the pJB12-scFv library and phages were prepared and titrated by determining colony-forming-units (CFU) as described (15, 27). For depletion of phage antibodies targeting antigens on healthy cells, 10^12^ phages were added to 10^8^ leukocytes in 2 ml PBS supplemented with 4% BSA (Carl Roth; cat. no. 8076.3). After 2 h on a roller incubator at 4°C, the cells were separated by centrifugation (1,900 x g, 10 min, 4°C) and depleted as described above with 10^8^ leukocytes from a second healthy donor. For selection, 2 x 10^6^ L-363 cells were resuspended in the supernatant containing non-depleted phages. After 30 min on a roller incubator at 4°C, the cells were washed five times using ice-cold PBS supplemented with 2% BSA and two times using ice-cold PBS. For elution of cell surface bound phages, cells were incubated with trypsin (1 mg/ml in PBS; Sigma-Aldrich; cat. no. T1426) for 10 min at room temperature and centrifuged (18,000 x g, 10 min). The supernatant containing the eluted phages was added to XL1 Blue *E. coli* (OD_600nm_ = 0.5, 10 μg/ml tetracycline) and incubated at 37°C for 30 min. Bacteria were plated on 2xYT agar plates containing 1% glucose, 30 μg/ml chloramphenicol and 10 μg/ml tetracycline to prepare phages for a second panning round as described above.

### Sequencing Analyses

Phagemids were isolated from infected *E. coli* using the NucleoBond Xtra Maxi Kit (Machery-Nagel; cat. no. 740414.50). Variable regions were amplified by PCR with forward primer placed 5’ of VH region and reverse primers binding J genes (**Supplementary Table 2**) as described in the **Supplementary Information**. PCR products were sequenced by Illumina MiSeq system using the MiSeq Reagent Kit v3 (Illumina) resulting in 2 x 300 nt reads. Clonotypes of IGHV-IGHD-IGHJ (hereon “VH”) rearrangements were identified using ARResT/Interrogate [arrest.tools/interrogate] and frequencies were calculated as percentage of reads with identified rearrangements (28). A clonotype was defined by its IGHV and IGHJ genes, the segmentation producing the junction (IGHV and IGHJ deletions and the length of the N-IGHD-N region), and the junction amino acid (aa) sequence itself [coding region from aa position 104 to 118 containing CDRH3, as defined by IMGT (29)]. As sample diversity calculations are influenced by sequencing depth, 50,000 sequences of each NGS data set were randomly selected and diversity as effective number of species (30) (counted in clonotypes, representing the number of equally abundant clonotypes needed for the average proportional abundance of the clonotypes to equal that observed in the sample, where all clonotypes may not be equally abundant; directly calculable from Shanon entropy), evenness of the distribution (scale 0 to 1; from Shanon entropy) (31) and dominance as percentage of the most frequent clone were calculated using in-house Python scripts. Phagemids of chosen candidates from the 2^nd^ panning round were Sanger sequenced to obtain the full-length scFv sequences.

### Production of Monoclonal Phage Antibodies

To prepare monoclonal phage antibodies, phagemid carrying *E. coli* were grown to an OD_600nm_ of 0.5 in 5 ml 2xYT medium supplemented with 1% glucose, 30 μg/ml chloramphenicol and 10 μg/ml tetracycline. After infection with M13KO7 helper phage (20x excess, 30 min, 37°C), medium was replaced with fresh medium containing 1% glucose, 30 μg/ml chloramphenicol, 0.5 mM IPTG, 25 µg/ml kanamycin and incubated for 16 h at 30°C. Phage-containing supernatants were separated by centrifugation (4°C, 3,350 x g, 20 min) and titers were estimated by enzyme-linked immunosorbent assay (ELISA) according to standard procedures (27).

### Whole Cell ELISA

96-well-plates were blocked overnight with PBS supplemented with 4% BSA. The following day, 1 x 10^6^ cells per well were blocked for 30 min on ice with PBS supplemented with 4% BSA. 100-150 µl bacterial supernatant containing approximately 1 x 10^9^ phages were mixed with the cells and incubated for 1 h at 4°C. CD7-targeting scFv phage was used as control (32). Plates were washed 3-times with cold PBS supplemented with 0.1% BSA and subsequently incubated for 1 h at 4°C with anti-M13-HRP antibody (Creative Diagnostics; cat. no. CAB-655M; diluted 1:2000 in PBS supplemented with 4% BSA). After washing as described above 100 µl ABTS solution/well (Roche; cat. no. 11112422001) was added. Absorbance at 405 nm was measured with Sunrise absorbance microplate reader (Tecan; reference wavelength 492 nm).

### Flow Cytometric Analyses

To analyze cell surface binding, 5 x 10^5^ cells were incubated with 10^11^ phages or IgG antibody at the indicated concentrations in PBS supplemented with 1% BSA and 0.1% NaN_3_. After 1 h at 4°C, phages were detected with anti-fd bacteriophage rabbit antibody (Sigma-Aldrich) and with donkey anti-rabbit IgG F(ab)_2_ fragment-FITC (Jackson Immuno Research; cat. no. 711-096-152), while IgG antibodies were detected with goat anti-human Fcγ F(ab)_2_ fragment–FITC (Jackson Immuno Research; cat. no. 109-096-098) or used directly labeled with DyLight755 Antibody Labeling Kit (Thermo Fisher Scientific; cat. no. 84539) according to manufacturer’s instructions. For blocking experiments, 5 µg/ml DyLight755-labeled CD38 antibodies were used after pre-incubation with 1 mg/ml unlabeled CD38 antibodies or control antibody. Samples were measured on a Navios flow cytometer and analyzed with Kaluza software 1.3 (Beckman Coulter).

### Generation of Chimeric #5-CD38-IgG1

Restriction sites for enzymes *Nhe*I and *PpuM*I or *Hind*III (all NEB; cat. no. R0131S, R0506S, R0104S) were integrated in the 5’ and 3’ of VH and VL sequences of phage antibody #5 and synthesized by Eurofins Genomics (Hamburg). VH and VL were cloned into modified pSEC vectors containing the human constant regions of IgG1 heavy chain (HC) and kappa light chain (LC), respectively, as previously described (33). The expression vectors (pSEC-VH#5-HC-IgG1 and pSEC-VL#5-LC-kappa) were prepared endotoxin-free by NucleoBond PC 2000 EF Mega kit (Machery-Nagel; cat. no. 740549) and final sequences were verified by Sanger sequencing. Chimeric #5-CD38-IgG1 antibody was produced by electroporation of 600 µg each pSEC-VH#5-HC-IgG1 and pSEC-VL#5-LC-kappa in CHO-S cells using the STX-100 System from MaxCyte and IgG1 antibody was purified from cell supernatants by affinity chromatography with CH1 matrix according to manufacturer’s introductions (Thermo Fisher Scientific; cat. no. 2943452010). Purity and concentration were analyzed by sodium dodecyl sulphate-polyacrylamide gel electrophoresis (SDS-PAGE) followed by Coomassie staining (Carl Roth; cat. no. A152.1; **Supplementary Figure S4**), and Pierce BCA Protein Assay (Thermo Fisher Scientific; cat. no. 23225). Daratumumab and isatuximab were purchased from the pharmacy of the University Hospital Schleswig-Holstein.

### Chromium Release Assay

Antibody-dependent cell-mediated cytotoxicity (ADCC) and complement-dependent cytotoxicity (CDC) were measured by chromium release assay as previously described for 4 h (34). Briefly, tumor cells were labeled with radioactive ^51^ ${\text{CrO}}_{4}^{2-}$ and incubated with healthy donors’ PBMC at an effector to target (E:T) cell ratio of 80:1 or 25% (v/v) human serum in the presence of the indicated antibodies. Percentage of lysis was calculated from counts per minute (cpm) as follows: % lysis = (experimental cpm x basal cpm)/(maximal cpm x basal cpm) x 100.

### Phagocytosis Assay

M0 macrophages expressing Fcγ receptors were generated from PBMC of healthy donors by incubation with monocyte-attachment medium (PromoCell; cat. no. C-28051) for 30 min at 37°C and subsequent cultivation in X-VIVO 15 medium (Lonza; cat. no. BEBP02-061Q) supplemented with 50 ng/ml M-CSF (PeproTech; cat. no. 300-25) (35). After 11-13 days, macrophages were washed with PBS and detached by using cell-dissociation buffer (Thermo Fisher Scientific; cat. no. 13151014). Tumor cells were labeled with pHrodo (Thermo Fisher Scientific; cat. no. P36600) according to manufacturer’s introductions and added at an E:T ratio of 1:1 to the macrophages. Antibodies were added at the indicated concentrations and antibody-dependent cellular phagocytosis (ADCP) was measured for 6 h at 37°C. Red counts per image were counted every 20 min and analyzed on an IncuCyte SX1 (Sartorius).

### Programmed Cell Death Assay

5 x 10^5^ cells were incubated with 1 µg/ml of the indicated antibody for 20 h. Subsequently, cells were washed twice with PBS supplemented with 1% BSA and 0.1% NaN_3_ and incubated with Annexin V-FITC and 7-AAD from BioLegend (cat. no. 640922) according to manufacturer’s introductions. Samples were measured on a Navios flow cytometer and programmed cell death (PCD) was calculated as percentage of Annexin V^+^ cells representing early and late apoptotic cells.

### CD38 Enzymatic Activity Assay

The inhibition of the enzymatic activity of CD38 was measured using the CD38 Inhibitor Screening Assay Kit (Cyclase Activity) from BPS Bioscience (cat. no. 71275). 25 nM of antibody were incubated with 50 nM recombinant CD38 at room temperature for 15 min. 60 µM nicotinamide guanine dinucleotide (NGD^+^) was added and fluorescence of produced cyclic GDP-ribose (cGDPR) was measured (Ex 300 nm, Em 410 nm) after 25 min. Percent inhibition were calculated compared to controls without antibody.

### Structural Analysis

Co-crystallization structures of daratumumab and isatuximab Fab fragments binding CD38 were merged by superposition of CD38 using ChimeraX 1.1 (36–38).

### Data Processing and Statistical Analyses

Data were analyzed with GraphPad Prism 5.0 (GraphPad Software Inc.). Group data are reported as mean ± SEM. Differences between groups were analyzed by two-way ANOVA with Tukey’s multiple comparisons test or two-tailed t-test. Curves were fitted using a nonlinear regression model with a sigmoidal dose response (variable slope). Significance was accepted with p < 0.05.

## Results

### Monitoring Cellular Panning of the Immune Library by NGS Revealed Enrichment of scFv Phage Antibodies After Two Rounds

**Figure 1:**
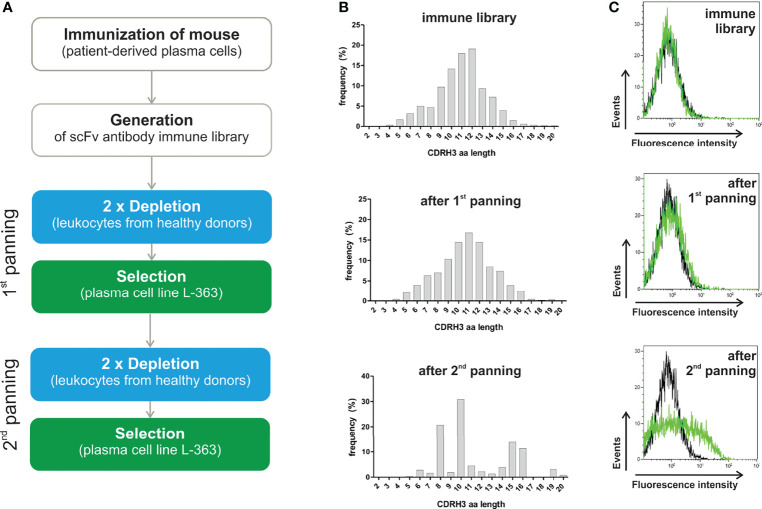
Panning strategy and results of NGS and flow cytometry analyses. **(A)** A scFv antibody library was generated from a mouse repeatedly immunized with PBMC from a PCL patient (95% malignant plasma cells) showing the highest titer of anti-plasma cell antibodies (refer to **Supplementary Figure S1**). Cellular panning to enrich myeloma binding phage antibodies were performed by two-times depletion with leukocytes (blue) and subsequent positive selection on L-363 plasma cell line (green), which was repeated two times. **(B)** Before and after each panning round, the scFv antibodies were analyzed by NGS to calculate diversity, evenness and dominance (**Table 1**) and define the frequency as % of the CDRH3 amino acid (aa) lengths. **(C)** In addition, flow cytometric analyses of the polyclonal phage antibody preparations (green) in comparison to a CD7 binding control phage (black) were performed with L‐363 cells to test for myeloma binding.

To generate a scFv antibody immune library, BALB/c mice were repeatedly immunized with PBMC from a PCL patient containing 95% malignant plasma cells. Total RNA was prepared from spleen of the mouse showing the highest antibody titer on human plasma cells (**Supplementary Figure S1**). V regions of the mouse antibodies’ heavy and light chains were amplified by PCR, randomly assembled to scFv and subsequently cloned into pJB12 phagemids (**Supplementary Figure S2**, for details please refer to **Supplementary Information**). By this, an immune library with 15 x 10^6^ colonies was generated from which 12 x 10^6^ CDRH3 amino acid (aa) sequences with 1.2 x 10^6^ unique CDRH3 aa sequences (1 of 10) were identified by NGS analyses (**Table 1**). The CDRH3 of the mouse immune library had a mean length of 12 aa and a bell-shaped distribution (**Figure 1B**). To gain more insights into the presence, frequency, evenness and dominance of single B cell clones, clonotypes were defined as unique combination of IGHV and IGHJ genes, segmentation and junction aa sequence generated during VDJ recombination. Within the immune library 1.9 x 10^6^ different VH clonotypes were identified with the most dominant one representing 2.35% of all sequences. Diversity was calculated to be 8,373 clonotypes representing the effective number of species from 50,000 randomly selected NGS sequences (**Table 1**). Evenness of the VH clonotype distribution was 0.89 meaning that the frequency of VH clonotypes in the immune library is – as expected – moderately uneven compared to a fully even distribution (evenness of 1), which can be expected for a synthetic antibody library with randomly mutated CDRs (19).

**Table 1:** Summary of sequencing analyses of the immune library during panning.

	Immune library	After 1^st^ panning	After 2^nd^ panning
**CDRH3 aa sequences**	12 x 10^6^	0.24 x 10^6^	0.82 x 10^6^
**Unique CDRH3 aa sequences**	1.2 x 10^6^	24,167	18,926
**VH clonotypes**	1.9 x 10^6^	0.19 x 10^6^	26,204
**Diversity**	8,373	4,492	95
**Evenness**	0.89	0.87	0.55
**Most frequent clonotype**	2.35%	1.60%	12.35%

To enrich myeloma binding antibodies, the immune library was depleted with leukocytes of healthy donors prior to positive selection on human myeloma cells in two consecutive rounds of panning (**Figure 1A**). After the first panning round, 0.19 x 10^6^ clonotypes with a 54% reduced diversity of 4,492 clonotypes as effective number of species was determined. Nevertheless, the relative amount of unique CDRH3 aa sequences (0.24 x 10^6^ total with 24,167 unique, 1 out of 10), the evenness (0.89 to 0.87) and the bell-shaped distribution of CDRH3 aa length remained (**Table 1**, **Figure 1B**). A clear change in the distribution of the CDRH3 aa lengths was seen after the second panning round, when predominantly CDRH3 aa lengths of 10 aa (accounting for 30.9%), 8 aa (20.7%), 15 aa (13.8%) and 16 aa (11.3%) were found (**Figure 1B**). NGS analyses revealed that 26,204 VH clonotypes were present with a 47-times reduced effective number of species (95 clonotypes) and 0.82 x 10^6^ CDRH3 aa sequences were composed of 18,926 unique CDRH3 aa sequences (1 out of 43) (**Table 1**). The most frequent VH clonotype was represented by 12.35% of sequences and evenness was considerably reduced to 0.55 (**Table 1**). These data indicate a successful elimination of undesired and an enrichment of desired antibodies through the applied cellular panning strategy after two panning steps. Of note, considerable binding to L-363 cells could be measured for the polyclonal phage antibodies by flow cytometry after the second panning round, whereas marginal myeloma binding was seen for the phages from the initial library and after the first panning round – nicely fitting to the NGS data (**Figure 1C**).

**Figure 2:**
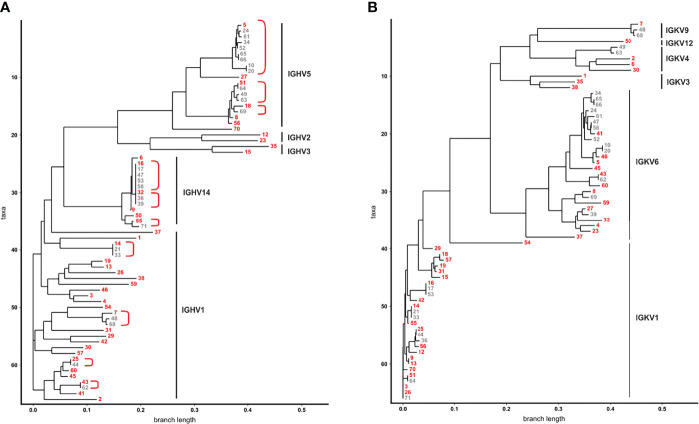
Dendrograms showing the similarity of VH and VL aa sequences from 66 phage antibodies after the second panning round. **(A)** VH and **(B)** VL of 66 scFv phage antibodies after the second panning round were Sanger sequenced and aligned by ClustalW using Neighbour Joining to represent similarities of aa sequences as dendrograms. The 66 VH aa sequences are represented by 41 VH clonotypes. For each of the 41 VH clones one member was chosen as a prototype (red) for further analysis. VH aa sequences sharing the same VH clonotype are grouped together in red brackets. The V gene subgroups are according to IMGT and are indicated on the right.

To get a more detailed picture of the scFv phage antibodies isolated after two rounds of panning, 66 randomly selected monoclonal phage antibodies were Sanger sequenced, their aa sequences of the VH and VL regions were aligned and summarized as dendrograms depicting aa sequence dissimilarity as branch length (**Figure 2**). 45 VH (68.2%) and 46 VL aa sequences (69.7%) were unique, while 21 (31.8%) and 20 aa sequences (30.3%), respectively, appeared repeatedly (**Figure 2**). For instance, the VH aa sequences of antibodies #5 and #24 were identical except from one aa, while #5 and #16 shared only 17.9% (21 of 117) aa (**Figure 2A**). Similar results were obtained for the VL aa sequences, where e.g. antibodies #16 and #17 were identical, while #5 and #16 shared only 51.8% (58 of 112 aa) of their VL aa sequence (**Figure 2B**).

As high similarity in aa sequences results from usage of closely related immunoglobulin (Ig) genes, V genes of the scFv phage antibodies were defined. Mice V genes of the heavy chain locus are grouped in 16 subgroups, dominated by IGHV1 and IGHV5 (39). These subgroups were also predominantly found [i.e. 30 IGHV1 (45.5%) and 19 IGHV5 subgroup (28.8%)] among the 66 monoclonal phage antibodies randomly selected after two rounds of panning (**Figure 2A**). Interestingly, 13 scFv phage antibodies (19.7%) contained VH genes of the genetically rather rare IGHV14 subgroup (**Figure 2A**). A dominance of these subgroups including IGHV14 could be already observed in our mouse immune library before panning with IGHV1, IGHV5 and IGHV14 accounting for 37%, 33% and 11% of the VH sequences, respectively. Taken together, 41 different VH clonotypes were identified among the 66 scFv phage antibodies (**Figure 2A**). Selection of scFv antibodies for binding analysis was predominantly done based on their VH clonotype since the VH is often substantially responsible for antigen binding and shows higher diversity (40). From the most dominant IGHV1 subgroup, 24 out of 30 (80%) antibodies represented individual VH clonotypes with CDRH3 lengths of 8 to 16 aa and were chosen for subsequent binding analysis (**Figure 2A**). In contrast, within the IGHV5 subgroup, 15 from 19 phage antibodies (78.9%) belonged to only three VH clonotypes with CDRH3 length of 10 and twice 16 aa, respectively. The overall most abundant VH clonotype (CDRH3 length of 10 aa) was found in 9 antibodies within this subgroup from which #5 was selected as a representative (**Figure 2A**). Six additional IGHV5 subgroup antibodies (#8, #18, #27, #51, #56, and #70) with CDRH3 lengths of 8 aa, 13 aa and predominantly 16 aa were chosen for further testing. Two antibodies from IGHV2 (#12 and #23 with CDRH3 aa lengths of 10 and 11, respectively) and IGHV3 (#15 and #35 with CDRH3 aa lengths of 10 and 15, respectively) subgroups were also selected for subsequent binding analyses due to their unique VH clonotypes (**Figure 2A**). The second overall most abundant VH clonotype was found in 5 antibodies and belonged to subgroup IGHV14 from which scFv antibody #16 (CDRH3 length of 8 aa) was selected for testing. Interestingly, the VH aa sequence of these 5 antibodies was identical to three other antibodies, but differed in 4 nucleotides in the CDRH3 leading to a unique VH clonotype represented by scFv antibody #32 (**Figure 2A**). Sanger sequencing revealed that the VH sequences of #16 and #32 were randomly combined to VL sequences belonging either to IGKV1 or IGKV6 in the scFv antibodies (**Figure 2B**). With 28 (42.4%) and 26 (39.4%) VL aa sequences, IGKV1 and IGKV6 subgroups represented the most dominant two subgroups found within the 66 phage antibodies. Of note, similar VL aa sequences from the IGKV6 subgroup can be observed for the 9 antibodies of the most dominant VH clonotype (represented by #5; IGHV5) potentially indicating an appropriate VH-VL pairing leading to enrichment of the respective scFv antibodies (**Figure 2B**). In contrast, the second most frequent VH clonotype (represented by #16; IGHV14) was found in combination with VL from either IGKV1 (#16, #17, #53) or IGKV6 (#47, #58) potentially indicating a higher flexibility in VH-VL paring for antigen binding of these scFv antibodies. In addition to IGKV1 and IGKV6, 12 VL aa sequences were identified to belong to IGKV3, IGKV4, IGKV9 and IGKV12 with at least one VL clonotype from each subgroup representatively present in the subsequently tested 41 scFv antibodies (**Figure 2B**).

### Selected Phage Antibodies Showed Preferential Binding to Myeloma Cells

**Figure 3:**
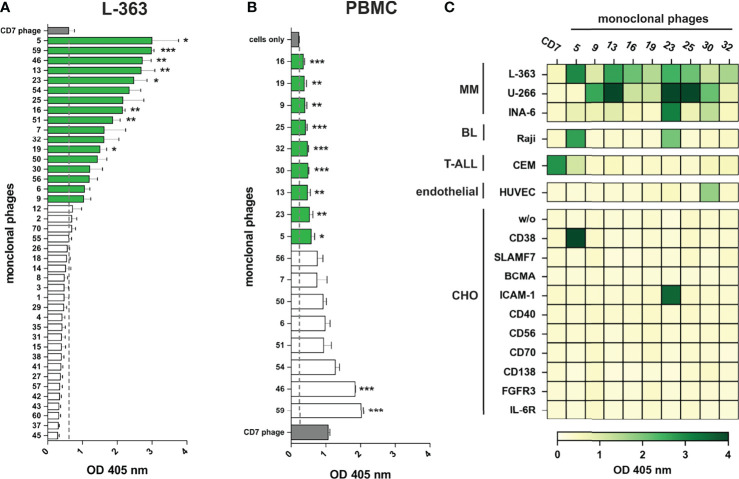
Binding analyses of monoclonal phage antibodies. **(A)** 1 x 10^6^ L‐363 myeloma cells were incubated with 1 x 10^9^ colony-forming-units (CFU) from the 41 different VH clonotypes and tested in ELISA experiments. Cell surface bound phages were detected using anti-M13-HRP antibody and absorbance was measured at 405 nm. 17 phages (green) showed a signal above the one obtained for CD7-specific phage (grey; dotted line), which was used as negative control. **(B)** Results of ELISA experiments performed equally with 1 x 10^6^ PBMC from healthy donors are shown. 9 phage antibodies (green) bind low to moderate to PBMC (green) compared to cells alone (grey; dotted line). CD7-specific phage (grey) showed antigen-dependent binding to T cells and NK cells and served as positive control in these experiments. Mean values ± SEM of three independent experiments are shown for these ELISA experiments. *p ≤ 0.05, **p ≤ 0.01, ***p ≤ 0.001 monoclonal phage vs. control. **(C)** The 9 most promising candidates were further tested in whole cell ELISA experiments with plasma cell lines (MM) L-363, U‐266, and INA-6, Raji Burkitt’s lymphoma (BL) cell line, T cell acute lymphoblastic leukemia cell line (T-ALL) CEM, human endothelial cells (endothelial) from healthy donors (HUVEC) and stable transfected CHO cells expressing either CD38, SLAMF7, BCMA, ICAM-1, CD40, CD56, CD70, CD138, FGFR3 or IL-6R. Color bar was set OD_405 nm_ = 0 (white) to OD_405 nm_ > 4 (dark green).

In total, 41 monoclonal phage antibodies representing the 41 individual VH clonotypes were tested in whole cell ELISA experiments with plasma cell line L-363, which was also used for panning. Seventeen antibodies showed substantial myeloma cell binding, which was taken as pre-requisite for further testing since therapeutic antibodies often require high antigen expression to mediate potent cytotoxicity against tumor cells (**Figure 3A**). Signal intensity varied between the scFv phage antibodies although identical 1 x 10^9^ phages were used, indicating various and differently expressed targeted antigens on L-363 cells (**Figure 3A**). Next, these 17 antibodies were tested for binding to PBMC from healthy donors to stringently select for myeloma-specific antibodies. Nine antibodies (#5, #9, #13, #16, #19, #23, #25, #30, #32) showed very little to moderate binding on blood cells, which was significantly weaker than that of the CD7 control phage antibody binding T cells and NK cells representing approximately 80% of the PBMC (**Figure 3B**).

The remaining 9 candidates were further analyzed for binding to plasma cell lines U‐266 and INA‐6, to Raji Burkitt’s lymphoma cell line and to T cell acute lymphoblastic leukemia cell line (T-ALL) CEM as well as HUVEC human endothelial cells. Importantly, all antibodies showed binding to at least two different plasma cell lines and therefore most likely recognize antigens expressed on myeloma cells. Only #30 was observed to bind to HUVEC endothelial cells (**Figure 3C**). Phage antibodies #5 and #23 bound to B and/or T cell lymphoma/leukemia cells and were identified as CD38 and ICAM-1 antibodies, respectively, by using stably transfected CHO cell lines expressing one out of 10 individual human myeloma-associated antigens (**Figure 3C**). Of note, none of the other 7 myeloma cell binding scFv phage antibodies showed reactivity to the CHO cell lines expressing MM antigens like BCMA, CD138 or SLAMF7, and might therefore recognize other, potentially novel antigens on myeloma cells.

### Selected Phage Antibodies Were Differently Enriched by Cellular Panning

To unravel how the myeloma binding scFv phage antibodies were enriched through panning, their sequences were tracked within the NGS data to determine their frequencies in the immune library and after the second panning round. Results are summarized in **Table 2** and show that all 9 antibodies were clearly enriched through the two rounds of panning. For instance, the CD38 binding scFv phage antibody #5 was already present with a high frequency of 0.045% in the immune library – potentially indicating a successful immunization – and was subsequently enriched to 9.6% (212-fold; **Table 2**) representing the second most frequent sequence after panning. A similar enrichment (202-fold) was observed for the ICAM-1 binding scFv phage antibody #23, although the initial (0.0024%) and final (0.49%) frequencies were much lower (**Table 2**). In contrast, the scFv antibody #30 was present with a high frequency of 0.03% in the immune library, but was enriched only 41-fold by panning. Since the immune library was generated from a mouse immunized with plasma cells of a PCL patient and panning was performed with L-363 myeloma cell line originally derived from another patient, this result may potentially be explained by differences in the expression level of the antigen recognized by antibody #30 on malignant plasma cells of different patients, leading to a rather weak or even no enrichment of individual antibodies from the immune library. Nevertheless, the remaining 6 scFv phage antibodies binding yet unknown antigens were enriched by 380- to 15,436-fold (**Table 2**).

**Table 2:** Frequencies, V genes and CDRH3/CDRL3 aa sequences of the 9 myeloma binding antibodies.

#	frequency (%)			VH		VL	
	Immune library	After 2^nd^ panning	Enrichment	V gene	CDRH3	V gene	CDRL3
25	0.000458	7.069747	15,436	IGHV1S81	ARKRYYAMDY	IGKV1-110	SQSTHVPLT
13	0.000171	1.976183	11,557	IGHV1S29	ARGLRGAMDY	IGKV1-110	SQSTHVPWT
32	0.002420	5.528296	2,284	IGHV14-3	ARRLRLDY	IGKV6-17	HQHYFTPLT
16	0.003009	6.514579	2,165	IGHV14-3	ARRLRLDY	IGKV1-110	SQSTHVPYT
19	0.000016	0.020615	1,288	IGHV1S29	ARRDRLDY	IGKV1-117	FQGSHVPRT
9	0.000287	0.109105	380	IGHV14-3	ARRRY	IGKV1-110	SQSTHVPWT
5 (CD38)	0.045049	9.558421	212	IGHV5-9-3	ARQKNGYVDY	IGKV6-23	QQYNTYPYT
23 (ICAM-1)	0.002436	0.493202	202	IGHV2-6-4	ARYGNYVPFDY	IGKV6-17	QQHYSTPYT
30	0.029513	1.219803	41	IGHV1-7	ARHYGNLFDY	IGKV4-68	QQWSSNPYT

Highest enrichment was seen for antibody #25 that had a very low frequency of 0.00046% in the immune library and became the third most frequent phage antibody (7.1%) after the second panning round. *Vice versa*, this strong enrichment potentially indicates that the antigen bound by antibody #25 is in contrast to the antigen of antibody #30 highly expressed on L-363 cells, which is indeed supported by the binding signals given by both antibodies in the cellular ELISA (**Figure 3C**). Interestingly, scFv antibodies #16 and #32, which have different VH clonotypes but identical VH aa sequences, had comparable frequencies in the immune library (0.002% and 0.003%, respectively) and enrichment factors of 2,284 (#32) and 2,165 (#16) further supporting that both may bind the same, still unknown antigen (**Table 2**).

In summary, among the 9 myeloma-binding antibodies identified so far, four carried VH genes from the IGHV1 subgroup, three from the IGHV14 subgroup and one each from the IGHV2 and the IGHV5 subgroup, all having CDRH3 lengths between 6 and 11 aa. Their VL genes mostly belonged to the IGKV1 and IGKV6 subgroups with one VL originating from the IGKV4 subgroup – all harboring complementary determining region 3 of the light chain (CDRL3) of 9 aa (**Table 2**).

### NGS Data Indicate Potential for Additional Myeloma Binding Antibodies

**Figure 4:**
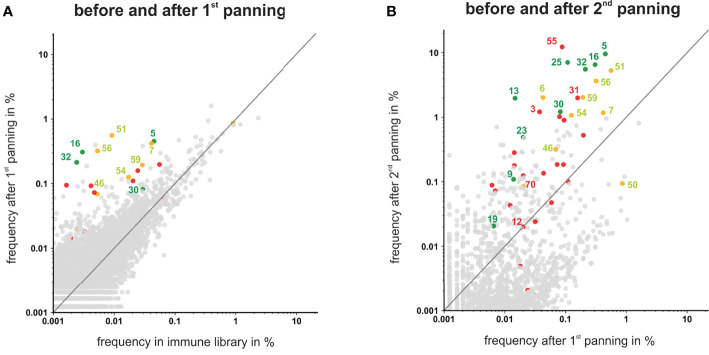
Scatter plots showing the frequencies of VH clonotypes after first and second panning. After the first **(A)** and second **(B)** panning VHs were sequenced by NGS, analyzed by ARResT/Interrogate and plotted. VH clones corresponding to scFv phages that showed binding to L-363 and only low or moderate binding to PBMC are labeled green. VH clones corresponding to scFv phages that bound to L-363, but were excluded due to PBMC binding, are labeled yellow. Candidates that showed no/low binding on L-363 are labeled red. Binding properties of the grey clones were not tested yet. VH clones depicted above the grey lines have a higher frequency in the library after panning than before.

To analyze if the 41 VH clonotypes from the randomly selected 66 colonies covered all highly enriched antibodies present in the library, frequencies of all VH clonotypes were plotted after each panning round (**Figure 4**). After the first panning round the majority of the VH clonotypes showed no significant enrichment, albeit some antibodies – including the myeloma restricted phage antibodies #5, #16, #32 and the myeloma and PBMC cross-reactive antibodies #7, #51, #56, #59 – were already enriched and present with a frequency >0.1% (**Figure 4A**). After the second panning round, these antibodies as well as the VH clonotypes of myeloma binding antibodies #25, #13, and #30 were further enriched and finally found with a frequency >1%. In addition, VH clonotypes of some antibodies excluded due to low/missing binding to L-363 myeloma cells (e.g. #3, #18, #31, #55) or cross-reactivity with PBMC (e.g. #6, #7) also showed clear enrichments with high frequencies >1% (**Figure 4B**). Antibodies like #55, the overall most frequent one after the two rounds of panning (12.3%), was not further characterized due to low binding to L-363 myeloma cells (**Figure 3A**). Such antibodies may actually bind low expressed antigens and may have therapeutic potential for highly efficient novel approaches like CAR T cells or bispecific antibodies, which do not require high antigen expression levels on tumor cells. Of note, the VH sequence of #55 belonged to the genetically rather rare IGHV14 Ig subgroup and had a CDRH3 length of 15 aa making this antibody even more interesting.

Taken together, by the random selection of 66 scFv phage antibodies, all antibodies present in the library with frequencies >1% after two rounds of panning were tested. Among the antibodies with frequencies between 1% and 0.1%, which can still be regarded as strongly enriched by the applied panning strategy, 42 antibodies were not tested yet and may include additional interesting candidates. Furthermore, the sequencing analyses revealed that antibodies like #50, which was the fourth most frequent clonotype in the initial immune library (0.9%), was finally depleted by 10-fold to a frequency of 0.09% by the applied panning on L-363 myeloma cells. Such antibodies may strongly bind to myeloma cells derived from other patients and can also be considered for further testing. Thus, combining phage display, cellular panning and deep sequencing is a suitable approach to allow the identification of novel antibodies against malignant plasma cells.

### Novel Chimeric CD38 Antibody #5-CD38-IgG1 Binds an Epitope Close to Daratumumab and Isatuximab and Has Potent Anti-Myeloma Activity

**Figure 5:**
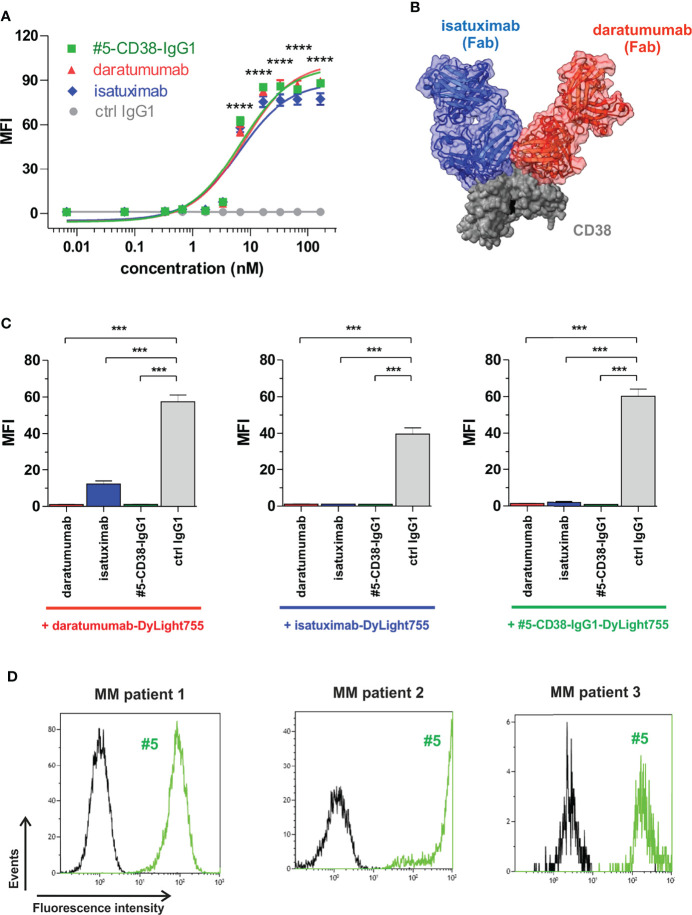
Binding analysis of chimeric antibody #5-CD38-IgG1. **(A)** L-363 cells were incubated with increasing concentrations of #5-CD38-IgG1 (green), daratumumab (red), isatuximab (blue) and control IgG1 (grey). Antibody binding to cell surface CD38 was detected by goat anti-human Fc F(ab)_2_ fragment-FITC and flow cytometry. Mean fluorescence intensity (MFI) ± SEM from three independent experiments are shown. ****p ≤ 0.0001 for CD38 antibodies vs. control IgG1. **(B)** Structures from co-crystallization of daratumumab Fab (red) and isatuximab Fab (blue) with the extracellular part of CD38 (grey, key residues of the catalytic active side indicated in black) merged by superposition of CD38 according references (36, 37). **(C)** To check for overlapping epitopes of #5-CD38-IgG1 with daratumumab or isatuximab on cell surface expressed CD38, 0.2 x 10^6^ L-363 cells were pre-incubated with 1 mg/ml #5-CD38-IgG1, daratumumab, isatuximab or control IgG1 prior incubation with 5 µg/ml DyLight755-labeled daratumumab (left graph), isatuximab (middle graph) or #5-CD38-IgG1 (right graph) and subsequently analyzed by flow cytometry. MFI ± SEM from three independent experiments are shown. ***p ≤ 0.001. **(D)** MNC from BM aspirates of three myeloma patients with 27% (left histogram), 4% (middle histogram) and 0.8% (right histogram) CD138-positive plasma cells, respectively, were checked for binding of FITC-labeled #5-CD38-IgG1 (#5, green) and corresponding control IgG1 antibody (black) by flow cytometry.

To investigate whether our newly identified anti-myeloma antibodies may have therapeutic potential, the VH and VL sequences of scFv phage antibody #5 were used to generate a chimeric CD38 IgG1 antibody for comparison with the clinically approved CD38 IgG1 antibodies daratumumab and isatuximab in first proof-of-concept studies. Affinity purification of #5-CD38-IgG1 antibody from CHO supernatants revealed highly pure protein showing the expected molecular weight of an IgG1 antibody (**Supplementary Figure S4**). Binding of #5-CD38-IgG1 to CD38-positive L-363 myeloma cells was compared to daratumumab and isatuximab by flow cytometric analyses with increasing antibody concentrations. Notably, #5-CD38-IgG1, daratumumab and isatuximab bound with similar affinity and almost equal half maximal (EC_50_ #5-CD38-IgG1 = 6.7 nM, EC_50_ daratumumab = 7.7 nM, EC_50_ isatuximab = 6.4 nM) and maximum binding to CD38 (**Figure 5A**). As shown for Fab fragments in **Figure 5B**, daratumumab and isatuximab bind to close, but distinct epitopes on CD38 on the opposite region of the catalytically active site (36, 37). Both antibodies bind epitopes so close to each other that steric hindrance and conformational changes of CD38 are induced that prevent binding of the other CD38 antibody, if one antibody is bound (36, 37). To analyze the binding epitope of #5-CD38-IgG1 in more detail, cross-blocking experiments with daratumumab and isatuximab on L‐363 cells were performed (**Figure 5C**). Isatuximab blocked 79% of CD38 binding of daratumumab, whereas daratumumab blocked 97% of isatuximab binding most likely due to the expected steric hindrance or conformational changes of the two antibodies upon CD38 binding. CD38 binding of #5-CD38-IgG1 was completely blocked by pre-incubation with daratumumab and isatuximab and *vice versa* – indicating close or overlapping epitopes of #5-CD38-IgG1, daratumumab and isatuximab (**Figure 5C**). Of note, #5-CD38-IgG1 also recognized CD38 on patient-derived malignant plasma cells derived from BM MNC of three myeloma patients (**Figure 5D**).

**Figure 6:**
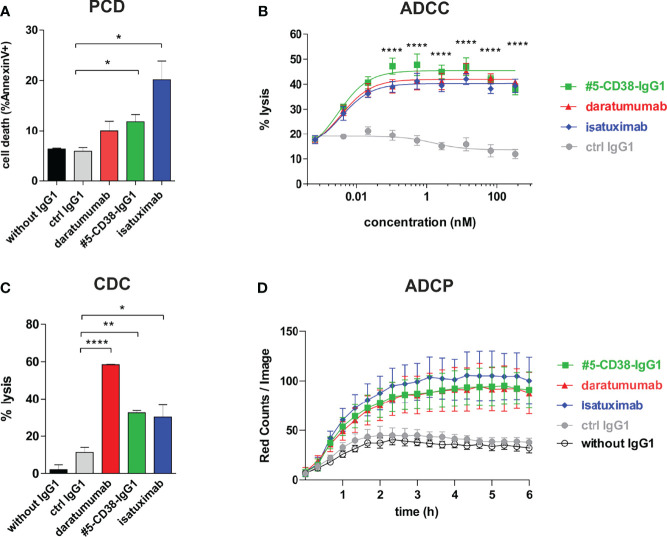
Functional characterization of #5-CD38-IgG1. **(A)** To measure the induction of PCD, 0.5 x 10^6^ Daudi cells were incubated with 1 µg/ml of the indicated antibodies for 20 h and afterwards stained with Annexin V-FITC and 7-AAD. Dead cells are shown as mean percentage of total Annexin V-positive cells ± SEM from three independent experiments. *p ≤ 0.05 CD38 antibodies vs. control IgG1. **(B)** ADCC was measured by standard 4 h chromium release assay using L-363 cells as target cells and PBMC of healthy donors as effectors cells in an effector-to-target cell (E:T) ratio of 80:1. Mean percentage of lysis ± SEM from six experiments with different PBMC donors are shown. ****p ≤ 0.0001 CD38 antibodies vs. control IgG1. **(C)** CDC was also measured by chromium release assay using 25% (v/v) serum of healthy donors and CD38-positive Daudi cells as targets. Mean percentage of lysis ± SEM from three independent experiments are shown. *p ≤ 0.05, **p ≤ 0.01, ***p ≤ 0.001 CD38 antibodies vs. control IgG1. **(D)** ADCP was measured by live cell imaging using pHrodo-labeled L-363 cells and macrophages generated from healthy donors in an E:T ratio of 1:1 with 10 µg/ml of the indicated antibodies. Phagocytosis was measured as red counts per image. Mean values ± SEM from four independent experiments are shown.

Although daratumumab and isatuximab both bind CD38, the antibodies have partly distinct modes of action, most likely due to slight differences in their binding epitopes. For instance, isatuximab is known to more efficiently induce programmed cell death (PCD) of myeloma cells and to inhibit the enzymatic activity of CD38 than daratumumab, while daratumumab in contrast to isatuximab potently induces CDC (36, 41–44). To evaluate the Fab- and Fc-mediated modes of action of #5-CD38-IgG1, we performed functional proof-of-concept studies. First, induction of PCD was analyzed by Annexin V staining after 20 h incubation of CD38-positive Daudi cells. As expected, isatuximab and #5-CD38-IgG1, but not daratumumab, significantly induced PCD of tumor cells when compared to control IgG1 (**Figure 6A**). Furthermore, #5-CD38-IgG1 was capable to inhibit 48% of the CD38 cyclase enzymatic activity, which lies in between the inhibition measured for isatuximab (69%) and daratumumab (22%) (**Supplementary Figure S5**). Next, to evaluate the potential of #5-CD38-IgG1 to recruit different components of the immune system for tumor cell lysis, standard chromium release assays with PBMC or serum of healthy donors and CD38-positive tumor cell lines were performed. As shown in **Figure 6B**, all three CD38 antibodies mediated significant ADCC of L-363 myeloma cells with comparable half-maximal (EC_50_ #5-CD38-IgG1 = 2.9 pM, EC_50_ daratumumab = 3.6 pM, EC_50_ isatuximab = 3.6 pM) and maximum killing (#5-CD38-IgG1 = 45.45 ± 1.31%, daratumumab = 41.97 ± 1.18%, isatuximab = 40.3 ± 0.95%; **Figure 6B**). In contrast, daratumumab was significantly more potent in inducing CDC than isatuximab and #5-CD38-IgG1 (**Figure 6C**). Daratumumab was capable to lyse 58.34 ± 0.27% of tumor cells, while isatuximab and #5-CD38-IgG1 showed significant and comparable tumor cell lysis of 30.13 ± 6.58% and 32.43 ± 1.24%, respectively (**Figure 6C**). Finally, ADCP activity of #5-CD38-IgG1 was analyzed with M0 macrophages expressing Fcγ receptors in live cell imaging assays, where phagocytosed myeloma cells were quantified as red counts per image over 6 h. All three CD38 antibodies mediated phagocytosis of myeloma cells to a comparable extent with maximum ADCP achieved within 3 h (**Figure 6D**).

In conclusion, #5-CD38-IgG1 bound with similar high affinity an overlapping epitope on CD38 and shared potent modes of action with the clinically approved CD38 antibodies daratumumab and isatuximab. Thereby, #5-CD38-IgG1 was capable to inhibit CD38 cyclase activity and to induce PCD, ADCC, CDC and ADCP comparable to daratumumab and isatuximab. Thus, these proof-of-concept studies revealed that a potent novel antibody could be isolated from our immune library.

## Discussion

By generating an immune library, using phage display, a cellular panning approach and deep sequencing, novel antibodies binding different antigens on malignant plasma cells could be isolated. One novel antibody targeted the well-established myeloma antigen CD38 and was converted into a chimeric IgG1. This antibody showed in first proof-of-concept studies potent anti-myeloma activity comparable to the clinically approved CD38 IgG1 antibodies daratumumab and isatuximab used for MM immunotherapy.

Today, myeloma patients are often treated with combination regimes including one of the monoclonal antibodies daratumumab, isatuximab or elotuzumab (3). Novel potent immunotherapeutic agents like ADCs, bispecific antibodies and CAR T cell products are clinically investigated (4, 5). Thereby, the majority of novel anti-myeloma immunotherapeutics target BCMA or CD38 on the cell surface of the malignant plasma cells (6). Unfortunately, still most of the patients eventually relapse – sometimes due to antigen loss of the tumor cells – highlighting an unmet medical need for novel potent immunotherapeutics and new antigens on myeloma cell surface (45, 46). To identify such antibodies, we immunized mice by 4 injections with patient-derived malignant plasma cells from one donor to generate matured, high-affinity antibodies and prepared a mouse immune library for phage display. A two-step cellular panning approach using leukocytes of healthy donors for depletion and human myeloma cell line L-363 for enrichment of myeloma cell surface binding antibodies was applied in accordance with published cellular panning strategies (8). Previously, such approaches were successfully used to identify tumor-specific antibodies like the anti-human C-type lectin-like molecule-1 antibodies for AML or anti-human ICAM-1 antibodies for MM therapy (8, 23, 47). ICAM-1 is an adhesion molecule highly expressed on myeloma cells and associated with drug-resistance in patients (48–50). Veitonmäki and colleagues identified an ICAM-1 antibody (BI-505) using cellular panning of a human phage antibody library, which was tested in a phase 1 trial for myeloma therapy (47, 51). BI-505 did not induce severe side effects, but lacked significant clinical anti-myeloma activity (51). In previous own work, we were able to show that Fc-engineering of an ICAM-1 antibody isolated from a synthetic human scFv antibody library markedly enhanced the antibody’s anti-myeloma activity *in vitro* and *in vivo* most likely by improving the recruitment of immune cells (24). That Fc-engineering can translate into clinically successful improvement of an antibody’s anti-tumor activity and may therefore also have potential to improve anti-myeloma activity of ICAM-1 antibodies was recently shown by the approval of tafasitamab-cxix, an Fc-engineered CD19 antibody for the treatment of diffuse large B cell lymphoma (52, 53). Interestingly, in this work we again isolated a novel ICAM-1 antibody (#23) from our immune library, but further investigation is needed to unravel the antibody’s characteristics and its potential for myeloma therapy.

In addition, we identified an antibody (#5) binding to CD38, a well-established antigen on myeloma cells and targeted by the clinically approved IgG1 antibodies daratumumab and isatuximab. CD38 is highly expressed on healthy and malignant plasma cells (54, 55). Daratumumab and isatuximab bind to close, but distinct CD38 epitopes, which translates into different modes of actions *in vitro*. While daratumumab is described to mediate potent CDC of CD38-expressing tumor cells and only slightly inhibiting CD38 enzymatic activity (42–44), isatuximab inhibits app. 80% of CD38 cyclase activity and induces more potent PCD than daratumumab that requires cross-linking *via* Fcγ receptors for PCD induction (36, 41, 43, 56). In contrast, both CD38 antibodies efficiently recruit immune cells for ADCC and phagocytosis of myeloma cells (36, 42, 57). To evaluate the therapeutic potential of our novel antibodies in first proof-of-concept studies, a chimeric #5-CD38-IgG1 was generated and tested in comparison to daratumumab and isatuximab. Binding analyses revealed that our novel #5-CD38-IgG1 binds an epitope close to the ones of both, daratumumab and isatuximab, and has comparable half-maximal binding to CD38 in the low nanomolar range indicating that high-affinity antibodies can be isolated by our approach. Further hint that #5-CD38-IgG1 recognizes a unique epitope is given by its characteristic to inhibit CD38 cyclase activity by app. 50%, which is actually in between the CD38 enzymatic inhibition reported for daratumumab and isatuximab (36, 44). Since CD38 is known to play an important role in the NAD^+^ metabolism and is believed to critically contribute to elevated adenosine levels in myeloma bone marrow niches (58), inhibition of the CD38 enzymatic activity and thereby disturbing the immunosuppressive microenvironment may be an important mechanism of action of #5-CD38-IgG1. Functional studies further revealed that #5-CD38-IgG1 was as potent as daratumumab and isatuximab in mediating ADCC and ADCP of CD38-positive tumor cells, while CDC activity was comparable to isatuximab and PCD induction was comparable to daratumumab. Thus, #5-CD38-IgG1 shares potent modes of actions with both clinically approved CD38 antibodies. These results demonstrate that potent high-affinity antibodies could be identified by our approach that may have potential as immunotherapeutics for MM. Of note, scFv antibodies #9, #13 and #25 showed strong binding to CD38-negative U266 and INA-6 myeloma cell lines and therefore may be especially interesting for patients’ refractory to CD38 immunotherapy.

NGS sequencing, which was performed at various time points to check the quality of the phage libraries, monitor the panning process and genetically characterize the antibodies based on their VDJ recombination, revealed that all of the so far identified myeloma-binding antibodies were highly enriched by panning. In line with this, Reddy and colleagues found target-specific antibodies with high frequency through sequencing of IGH and IGK regions from plasma cells of mice immunized with recombinant proteins (59). Using the Illumina MiSeq allowed covering the whole library and enabled the annotation of VH clonotypes, classification of V genes and determination of CDRH3 aa sequences. NGS data obtained for our scFv antibody library generated from a mouse immunized with patient-derived malignant plasma cells indicated that the 13 x 10^6^ VH nucleotide sequences encoded for 12 x 10^6^ CDRH3 aa sequences with 1.2 x 10^6^ unique CDRH3 aa sequences (10%). In contrast, synthetic and semi-synthetic antibody libraries miss the pre-selection by a natural immune system and have to have much higher diversity with up to 99% unique CDRH3 aa sequences to enable identification of desired antibodies (60). In line with previous works that required 2 to 3 panning rounds for cell-based selection of tumor-associated antibodies (8, 23), our NGS data showed a decrease in diversity and clear enrichment of individual phage antibodies from the immune library after two rounds of cellular panning. The bell-shaped distribution of the CDRH3 aa length, which had the expected mean of 11 to 12 aa in the immune library and after the first panning (61), shifted to predominant CDRH3 aa lengths of 10 aa, 8 aa, 15 aa and 16 aa after the second panning.

Tracking VH clonotypes representing antibodies derived from individual B cells showed that some of the later identified antibodies were already present at high frequency in the immune library and strongly enriched by the applied panning strategy, while the frequency of other antibodies decreased. This potentially indicates a successful enrichment of desired anti-myeloma antibodies. Furthermore, some antibodies finally identified as myeloma binders were initially very rare (<0.0005%) in the immune library and could be enriched by more than 10,000-fold. Interestingly, by randomly selecting 66 phage antibodies, all antibodies with frequencies >1% after two rounds of panning could be found, while 42 antibodies with frequencies between 0.1% and 1% were according to the NGS data not tested yet. These antibodies may still include interesting candidates, since for instance the myeloma binding antibodies #9 and #19, targeting potentially novel, yet unknown antigens, had frequencies of 0.1% and 0.02%, respectively. Recently, Ljungars and colleagues demonstrated that new tumor-restricted antibodies against chronic lymphocytic leukemia cells present with low frequencies after cellular panning in semi-synthetic antibody libraries (n-CoDeR) can successfully be identified by deep mining with NGS analyses (20). Thereby, NGS data can enable rescue of rare clones by PCR using specific primers and thus can help to bail out the full antibody repertoire present in phage display libraries (22, 60, 62). *Vice versa*, NGS data also enabled the identification of highly frequent antibodies that were excluded from further analyses due to low binding to L-363 myeloma cells in the initial ELISA experiments. Antibodies like #55, which was together with the CD38 binding antibody #5 the most frequent antibody (≥10%) after two rounds of panning, may target low expressed antigens on myeloma cells, which can be – comparable to BCMA – suitable targets for highly potent immunotherapeutic approaches (4, 5).

In conclusion, by combining phage display, cellular panning and NGS analyses of an immune library, we successfully identified two novel CD38 and ICAM-1 antibodies, and at least 7 antibodies targeting yet unknown, potentially novel antigens on myeloma cell surface. First proof-of-concept studies performed to investigate the therapeutic potential of our newly identified antibodies revealed that #5-CD38-IgG1 had comparable affinity and potent anti-myeloma activity to the clinically approved CD38 antibodies daratumumab and isatuximab. These data indicate that potent novel immunotherapeutics for MM therapy could be isolated by our approach.

## Data Availability Statement

The original contributions presented in the study are included in the article/**Supplementary Material**. Further inquiries can be directed to the corresponding author.

## Ethics Statement

The studies involving human participants were reviewed and approved by Ethics Committee of the Christian-Albrechts-University, Kiel, Germany. The patients/participants provided their written informed consent to participate in this study. The animal study was reviewed and approved by Christian-Albrechts-University, Kiel, Germany.

## Author Contributions

SK and KK: study design and data analyses. SK, AB, CG, and SL: conducting experiments, data acquisition and analyses. HK, DH, and ND: NGS and bioinformatics analyses. KW: recruitment of MM patients. SK and KK: took the lead in writing the manuscript in consultation with AS, MG, MB, and MP. All authors contributed to the article and approved the submitted version.

## Funding

This study was supported by a research grant from the Else Kröner-Fresenius-Stiftung (2015_A166) to KK and a research grant from the Deutsche Krebshilfe e.V. to MP.

## Conflict of Interest

The authors declare that the research was conducted in the absence of any commercial or financial relationships that could be construed as a potential conflict of interest.

## Publisher’s Note

All claims expressed in this article are solely those of the authors and do not necessarily represent those of their affiliated organizations, or those of the publisher, the editors and the reviewers. Any product that may be evaluated in this article, or claim that may be made by its manufacturer, is not guaranteed or endorsed by the publisher.

## Abbreviations

ADC, antibody-drug conjugate; ADCC, antibody-dependent cell-mediated cytotoxicity; ADCP, antibody-dependent cellular phagocytosis; BCMA, B cell maturation antigens; BM, bone marrow; BMSC, bone marrow stromal cells; CAR, chimeric antigen receptor; CDC, complement-dependent cytotoxicity; CDRH3, complementary determining region 3 of the heavy chain; CDRL3, complementary determining region 3 of the light chain; CFU, colony-forming units; ELISA, enzyme-linked immunosorbent assay; ICAM-1, intercellular adhesion molecule-1; Ig, immunoglobulin; MM, multiple myeloma; NGS, next-generation sequencing; PBMC, peripheral blood mononuclear cells; PCL, plasma cell leukemia; scFv, single-chain Fragment variable; VH, variable heavy chain; VL, variable light chain.
