# Supplementary material for: Identification of New Antibodies Targeting Malignant Plasma Cells for Immunotherapy by Next-Generation Sequencing-Assisted Phage Display
Source: Front Immunol. 2022 Jun 17;13:908093. doi: 10.3389/fimmu.2022.908093 (PMC9248769; doi:10.3389/fimmu.2022.908093)
Supplement: Supplementary file 1 [file DataSheet_1.pdf]

Supplementary Material

1 Supplementary material and methods

1.1 Flow cytometry analysis of serum from immunized mice

Mouse anti-myeloma antibody titer in final sera (1:1000) of BALB/c mice repeatedly immunized with PBMC containing 95 % malignant plasma cells from a PCL patient was measured by flow cytometry using cells from the same patient and sheep anti-mouse IgG F(ab')2 fragment-FITC (Sigma-Aldrich; cat. no. F2883-1ML). Sera from non-immunized mice were taken as control (**supplementary figure S1**).

1.2 Amplification of mouse V regions and generation of the mouse immune library

Mouse VH and light VL regions were amplified from spleen-derived cDNA by PCR using degenerated primer mixes binding mouse V and J genes (**supplementary table 1**). For PCR reactions 5 µl cDNA, 29 µl RNAase and DNAse-free water, 5 µl 10x *Pwo* reaction puffer, 1 µl dNTP mix (each 10 mM; Roche; cat. no. 4638956001), 4 µl $MgSO_4$ (25 mM), 2 µl DMSO, 1 µl forward primer mix (100 µM), 1 µl reverse primer mix (100 µM) and 2 µl polymerase mix (*Pwo* and *Taq* polymerase 5:1; VWR; cat. no. 01-5010, PEQL01-1010) were used. Amplification was started with five cycles of gradient PCR (1 min at 92°C, 1 min at 45-55°C, 1 min at 72°C) followed by 25 cycles with fixed annealing temperature (1 min at 92°C, 1 min at 63°C, 1 min at 72°C). The approximately 400 bp VH and VL fragments were separated by agarose gel electrophoresis and purified by gel extraction using the QIAquick kit from Qiagen (cat. no. 28706) according to manufacturer's introductions (**supplementary figure S2 A**). The PCR products and pUC19-MCS17 (derivate of pUC19, produced in our laboratory; **supplementary figure S3**) were digested with 1 U *Sfi*I restriction enzyme (NEB; cat. no. R0123L), purified by agarose gel electrophoresis and assembled using T4 ligase from NEB (cat. no. M0202T) according to manufacturer's introductions. The reactions were treated with 10 volumes n-butanol (Carl Roth; cat. no. 7724.1) and centrifuged (25,000 g, 5 min). The precipitates were washed two times with 70% ethanol and dissolved in water. XL1 Blue *E. coli* (Agilent; cat. no. 200228) were transformed with de-salted ligation reactions using the MicroPulser electroporator (Bio-Rad; cat. no. 1652100) according to manufacturer's introductions and bacteria were afterwards plated by using standard procedures. Plasmids representing the VH and VL sub-libraries were prepared from bacteria using the NucleoBond Xtra Maxi Kit from Machery Nagel (cat. no. 740414.50). Both sub-libraries (pUC19-MCS2017-VH and pUC19-MCS2017-VL) were digested using restriction enzymes *Asc*I und *Not*I-HF (NEB; cat. no. R0558S, R3189S) and purified by gel extraction (**supplementary figure S2 C**). The resulting VL fragments were randomly inserted into pUC19-MCS2017-VH as described above to generate pUC19-MCS2017-scFv. ScFv were cloned into pJB12 phagemids containing pIII gene of M13 filamentous phage by digestion with *Sfi*I restriction enzyme and transformed in XL1 Blue *E. coli* as described above (**supplementary figure S2 D**).(1) The XL1 Blue *E. coli* pJB12-scFv were suspended in 2YT medium containing 20% glycerol, snap frozen as aliquots, stored at -80°C and represent the mouse immune library.

1.3 Amplification of VH region for Illumina MiSeq

VH regions were amplified by PCR for adding adapters and indexes using EagleTaq DNA Polymerase (Roche; cat. no. 05206952190) according to manufacturer's instructions. The forward primer was placed 5' of VH region and reverse primers were a mix of J gene binding primers (**supplementary table 2**). PCR products were separated by agarose gel electrophoresis and approximately 400 bp PCR

products were purified from gel using Qiagen Min Elute Gel Extraction Kit (cat. no. 28606) according to manufacturer's instructions. Concentration and average fragment length of the PCR product were measured by Qubit fluorometer (Thermo Fisher Scientific) and Agilent 2100 Bioanalyzer, respectively.

2 References

1. Burmester J, Plückthun A. Construction of Scfv Fragments from Hybridoma or Spleen Cells by Pcr Assembly. In: Kontermann R, Dübel S, editors. *Antibody Engineering*. Berlin, Heidelberg: Springer Berlin Heidelberg (2001). p. 19-40.

Supplementary Table 1. Primer used for V region PCR to generate the mouse immune library. Annealing regions of primer for mouse V (VH for and VL for) and J (VH rev and VL rev) genes with degenerated bases were taken from Burmester *et al.*(1) Restriction sites (*Sfi*I in red boxes and *Not*I in blue boxes) and linker sequences (underlined) were added to 5' ends of the primer to allow final cloning to scFv's according to Material and Methods. R = A, G; Y = C, T; M =A, C; K = G, T; S = C, G; W = A, T; H = A, C, T; B = C, G, T; V = A, C, G; D = A, G, T

Name	Sequenz (5' → 3')
**VH for**	TTACTCGCGGCCCAGCCGGCCATGGCGGAKGTRMAGCTTCAGGAGTC
	TTACTCGCGGCCCAGCCGGCCATGGCGGAGGTBCAGCTBCAGCAGTC
	TTACTCGCGGCCCAGCCGGCCATGGCGCAGGTGCAGCTGAAGSARTC
	TTACTCGCGGCCCAGCCGGCCATGGCGGAGGTCCARCTGCAACARTC
	TTACTCGCGGCCCAGCCGGCCATGGCGCAGGTYCAGCTBCAGCARTC
	TTACTCGCGGCCCAGCCGGCCATGGCGCAGGTYCARCTGCAGCARTC
	TTACTCGCGGCCCAGCCGGCCATGGCGCAGGTCCACGTGAAGCARTC
	TTACTCGCGGCCCAGCCGGCCATGGCGGAGGTGAASSTGGTGGARTC
	TTACTCGCGGCCCAGCCGGCCATGGCGGAVGTGAWGSTGGTGGAGTC
	TTACTCGCGGCCCAGCCGGCCATGGCGGAGGTGCAGSTGGTGGARTC
	TTACTCGCGGCCCAGCCGGCCATGGCGGAKGTGCAMCTGGTGGARTC
	TTACTCGCGGCCCAGCCGGCCATGGCGGAGGTGAAGCTGATGGARTC
	TTACTCGCGGCCCAGCCGGCCATGGCGGAGGTGCARCTTGTTGARTC
	TTACTCGCGGCCCAGCCGGCCATGGCGGARGTRAAGCTTCTCGARTC
	TTACTCGCGGCCCAGCCGGCCATGGCGGAAGTGAARSTTGAGGARTC
	TTACTCGCGGCCCAGCCGGCCATGGCGCAGGTTACTCTRAAASARTC
	TTACTCGCGGCCCAGCCGGCCATGGCGCAGGTCCAACTVCAGCARCC
	TTACTCGCGGCCCAGCCGGCCATGGCGGATGTGAACTTGGAASARTC
	TTACTCGCGGCCCAGCCGGCCATGGCGGAGGTGAAGGTCATCGARTC
**VH rev**	TTACTCGCGGCCCCCGAGGCCGCGGCCGCCACCACCAGAACCACCACCACCCGAGGAAACGGTGACCGTGGT
	TTACTCGCGGCCCCCGAGGCCGCGGCCGCCACCACCAGAACCACCACCACCCGAGGAGACTGTGAGAGTGGT
	TTACTCGCGGCCCCCGAGGCCGCGGCCGCCACCACCAGAACCACCACCACCCGCAGAGACAGTGACCAGAGT
	TTACTCGCGGCCCCCGAGGCCGCGGCCGCCACCACCAGAACCACCACCACCCGAGGAGACGGTGACTGAGGT
**VL for**	TTACTCGCGGCCCAGCCGGCCGGCGGCCGCGGCGGCGGCGGCTCCGAYATCCAGCTGACTCAGCC
	TTACTCGCGGCCCAGCCGGCCGGCGGCCGCGGCGGCGGCGGCTCCGAYATTGTTCTCWCCCAGTC
	TTACTCGCGGCCCAGCCGGCCGGCGGCCGCGGCGGCGGCGGCTCCGAYATTGTGMTMACTCAGTC
	TTACTCGCGGCCCAGCCGGCCGGCGGCCGCGGCGGCGGCGGCTCCGAYATTGTGYTRACACAGTC
	TTACTCGCGGCCCAGCCGGCCGGCGGCCGCGGCGGCGGCGGCTCCGAYATTGTRATGACMCAGTC
	TTACTCGCGGCCCAGCCGGCCGGCGGCCGCGGCGGCGGCGGCTCCGAYATTMAGATRAMCCAGTC
	TTACTCGCGGCCCAGCCGGCCGGCGGCCGCGGCGGCGGCGGCTCCGAYATTCAGATGAYDCAGTC
	TTACTCGCGGCCCAGCCGGCCGGCGGCCGCGGCGGCGGCGGCTCCGAYATYCAGATGACACAGAC
	TTACTCGCGGCCCAGCCGGCCGGCGGCCGCGGCGGCGGCGGCTCCGAYATTGTTCTCAWCCAGTC
	TTACTCGCGGCCCAGCCGGCCGGCGGCCGCGGCGGCGGCGGCTCCGAYATTGWGCTSACCCAATC
	TTACTCGCGGCCCAGCCGGCCGGCGGCCGCGGCGGCGGCGGCTCCGAYATTSTRATGACCCARTC
	TTACTCGCGGCCCAGCCGGCCGGCGGCCGCGGCGGCGGCGGCTCCGAYRTTKTGATGACCCARAC
	TTACTCGCGGCCCAGCCGGCCGGCGGCCGCGGCGGCGGCGGCTCCGAYATTGTGATGACBCAGKC
	TTACTCGCGGCCCAGCCGGCCGGCGGCCGCGGCGGCGGCGGCTCCGAYATTGTGATAACYCAGGA
	TTACTCGCGGCCCAGCCGGCCGGCGGCCGCGGCGGCGGCGGCTCCGAYATTGTGATGACCCAGWT
	TTACTCGCGGCCCAGCCGGCCGGCGGCCGCGGCGGCGGCGGCTCCGAYATTGTGATGACACAACC
	TTACTCGCGGCCCAGCCGGCCGGCGGCCGCGGCGGCGGCGGCTCCGAYATTTTGCTGACTCAGTC
	TTACTCGCGGCCCAGCCGGCCGGCGGCCGCGGCGGCGGCGGCTCCGATGCTGTTGTGACTCAGGAATC
**VL rev**	TTACTCGCGGCCCCCGAGGCCGCACGTTTKATTTCCAGCTTGG
	TTACTCGCGGCCCCCGAGGCCGCACGTTTTATTTCCAACTTTG
	TTACTCGCGGCCCCCGAGGCCGCACGTTTCAGCTCCAGCTTGG
	TTACTCGCGGCCCCCGAGGCCGCACCTAGGACAGTCAGTTTGG

Supplementary Table 2. Primer used for VH region PCR for Illumina MiSeq. Forward primer (NGS for) was placed 5' of VH region on pJB12. Annealing regions of reverse primers for J genes (NGS rev) were taken from Burmester *et al.*(1) Adapter and indexes (underlined) were added to 5' ends of primes for Illumina MiSeq.

Name	Sequenz (5' → 3')
**NGS for**	AATGATACGGCGACCACCGAGATCTACACCAGGACGTACACTCTTTCCCTACACGACGCTCTTCCGATCT<br>CGGCAGCCGCTGGATTGTTATTAC
**NGS rev**	CAAGCAGAAGACGGCATACGAGATCGAGTAATGTGACTGGAGTTCAGACGTGTGCTCTTCCGATCT<br>CGAGGAAACGGTGACCGTGGT<br>CAAGCAGAAGACGGCATACGAGATCGAGTAATGTGACTGGAGTTCAGACGTGTGCTCTTCCGATCT<br>CGAGGAGACTGTGAGAGTGGT<br>CAAGCAGAAGACGGCATACGAGATCGAGTAATGTGACTGGAGTTCAGACGTGTGCTCTTCCGATCT<br>CGCAGAGACAGTGACCAGAGT<br>CAAGCAGAAGACGGCATACGAGATCGAGTAATGTGACTGGAGTTCAGACGTGTGCTCTTCCGATCT<br>CGAGGAGACGGTGACTGAGGT

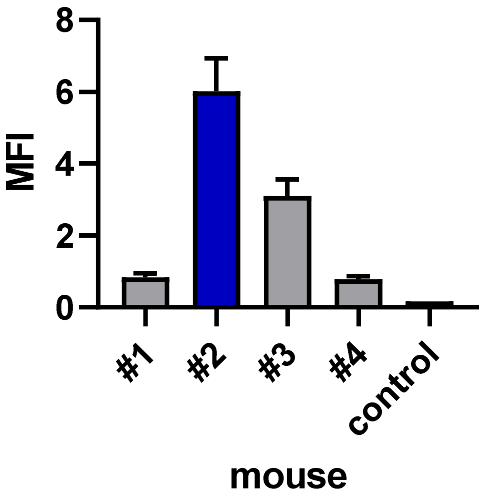

Supplementary Figure S1: Anti-plasma cell antibody titer of immunized mice. (a) Final sera (dilution 1:1000) of BALB/c mice immunized four times intraperitoneally with $1x10^6$ PBMC of a PCL patient containing 95% malignant plasma cells were tested by flow cytometry using 500.000 cells from the same patient/sample. Binding of the polyclonal mouse anti-plasma cell serum antibodies was detected by FITC-labelled anti-mouse IgG secondary antibody. As control, one mouse injected with PBS was used. The spleen of the mouse with the highest anti-plasma cell antibody titer (#2, blue) was taken to generate the immune library. Mean fluorescence intensity (MFI) ± SEM of two independent measurements is shown.

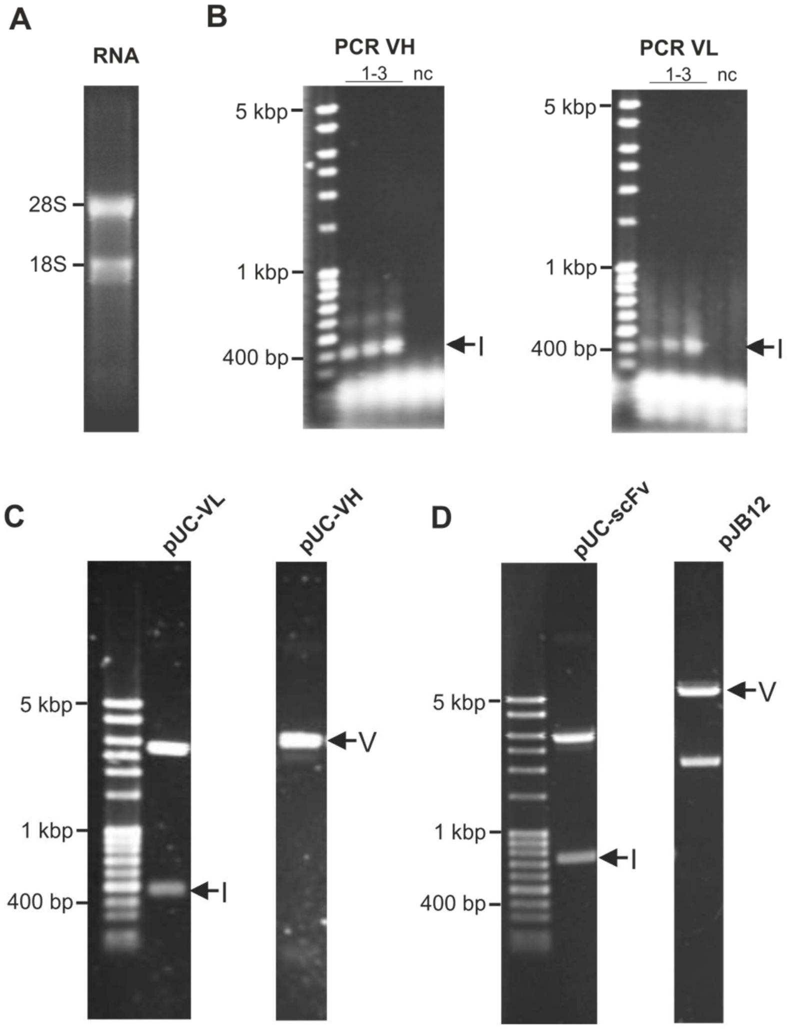

Supplementary Figure S2: Generation of immune library from total RNA. **(A)** Quality of total RNA from mouse spleen was controlled by agarose-formaldehyde gel electrophoresis. The two bands can be assigned to ribosomal RNA (28S and 18S) confirming integrity. **(B)** cDNA prepared from total RNA was used as template for amplification of mouse VH and VL regions by gradient PCR (annealing temperatures 1-3: 45°C, 50°C, 55°C; negative control (nc) without templates). The approximately 400 bp fragments were purified and used as insert (I) for further cloning in pUC19-MCS2017 to generate pUC19-MCS2017-VH (pUC-VH) and pUC19-MCS2017-VL (pUC-VL) sub-libraries. **(C)** These sub-libraries were digested using restriction enzymes *Asc*I und *Not*I-HF and separated by agarose gel electrophoresis to subsequently clone the approximately 500 bp VL fragments (I) into the linearized pUC19-MCS2017-VH plasmids (V). **(D)** The pUC-MCS2017-scFv plasmids were finally digested using restriction enzyme *Sfi*I to subclone the approximately 800 bp scFv fragments (I) into pJB12 phagemids (V). kbp: kilobase pairs; bp: base pairs

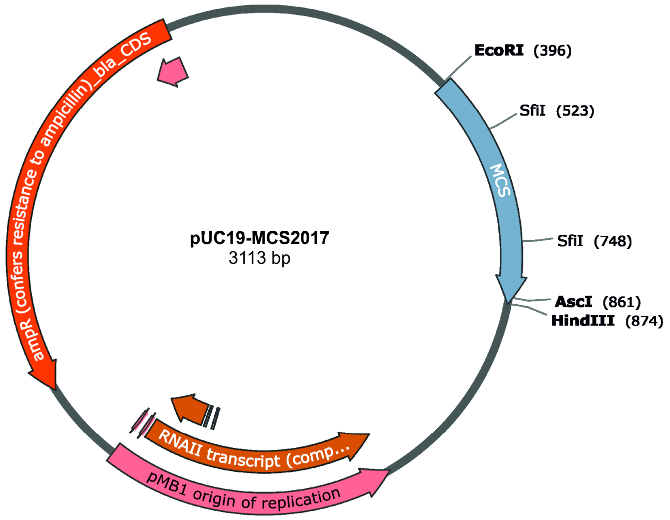

<u>gaattc</u>ttaattaatctagaaaacaaactgcact
tgttgagcttgtgaaacacaagcccaaggcaaca
aaagagcaactgaaagctgttatggatgatttcg
cagcttttgtagagaagt**ggcccagccggcc**tat
cttcagcagtgtccatttgaagatcatgtaaaat
tagtgaatgtgaatttgcaaaaacatgtgtagct
gatgagtcagctgaaaattgtgacaaatcacttc
atacccttttttggagacaaattatgcacagttgc
aactcttcgtgaaacctatggtgaaatggctgac
tgctgtgcaaaacaagaaccaattgatctttcgg
gggcc**ggcctcgggggcc**gctgcaaggctgacga
taaggagacctgctttgccgaggagggtaaaaaa
cttgttgctgcaagtcaagctgccttaggcttag
gtggtggcggcagttcctcgag**ggcgcgcc**gtta
ac<u>aagctt</u>

Supplementary Figure S3. Map of plasmid pUC19-MCS2017. The new multiple cloning site (MCS) that was added to pUC19 by *Eco*RI and *Hind*III (underlined) contains two *Sfi*I sites (red boxes) for inserting products from V region PCRs. The additional *Asc*I site (blue box) in the MCS and the *Not*I sites on the VH and VL PCR products are used for assembling scFv from pUC-MCS2017-VH and pUC-MCS2017-VL sub-libraries.

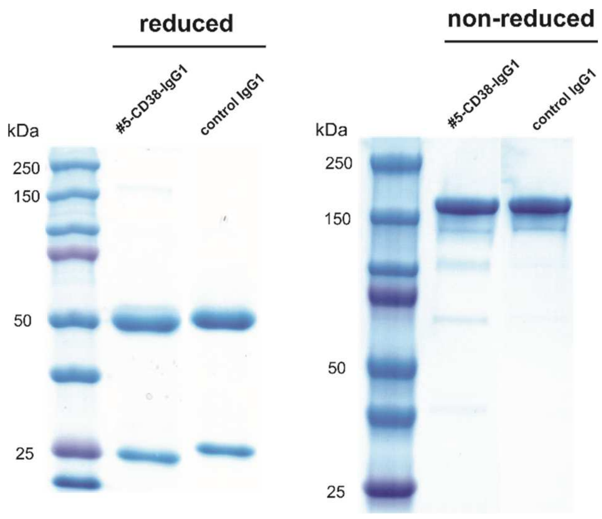

Supplementary Figure S4: SDS-PAGE and Coomassie staining of #5-CD38-IgG1 antibody. To determine molecular weight and purity of #5-CD38-IgG1, 6 µg protein were simultaneously analyzed with 6 µg of a control IgG1 by SDS-PAGE under reducing (left) and non-reducing conditions (right) followed by Coomassie staining. #5-CD38-IgG1 showed the expected molecular weight of approximately 50 kDa (heavy chain) and 25 kDa (light chain) under reducing conditions and 150 kDa (IgG1κ) under non-reducing conditions, respectively, comparable to the control IgG1κ. No contaminants could be observed.

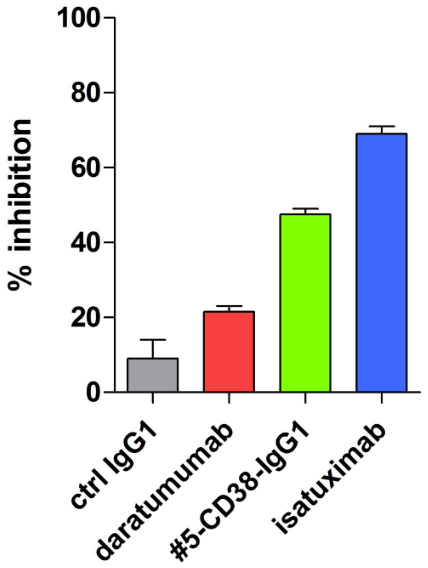

Supplementary Figure S5: Impact of #5-CD38-IgG1 antibody on CD38 enzymatic activity. To determine the impact of #5-CD38-IgG1 on the cyclase activity of CD38, 25 nM of the respective CD38 antibody were incubated with 50 nM recombinant CD38 at room temperature for 15 min. 60 µM nicotinamide guanine dinucleotide (NGD^+) was added and fluorescence of produced cyclic GDP-ribose (cGDPR) was measured (Ex 300 nm, Em 410 nm) after 25 min. Percent inhibition were calculated compared to controls without antibody. #5-CD38-IgG1 inhibits 48 % of the CD38 enzymatic activity, which is in between the inhibition observed with isatuximab (69 %) and daratumumab (22 %). Mean values ± SEM of two independent experiments are shown.
